# Supplementary material for: Evoked and transmitted culture models: Using bayesian methods to infer the evolution of cultural traits in history
Source: PLoS One. 2022 Apr 7;17(4):e0264509. doi: 10.1371/journal.pone.0264509 (PMC8989295; doi:10.1371/journal.pone.0264509)
Supplement: S1 Appendix — (PDF) [file pone.0264509.s001.pdf]

Supplementary Information to Manuscript: Evoked and Transmitted Culture models: Using bayesian methods to infer the evolution of cultural traits in history

Alexandre Hyafil,[1,*], Nicolas Baumard[2]
* alexandre.hyafil (at) gmail.com

Appendix A Fitting binary ETC model

A.1 E-step for binary traits

In the case of binary traits, exact inference can be performed using the standard forward-backward algorithm, irrespective of the model of artifact production. The algorithm yields the log-likelihood for current parameters $\boldsymbol{\theta}$. In the E-step, we compute the probability mass functions for the trait value and transitions given current values of the parameters [1, 2], i.e.

$\boldsymbol{\gamma}_t(\boldsymbol{\varepsilon}) = p(\boldsymbol{T}_t{=}\boldsymbol{\varepsilon}|\boldsymbol{A};\boldsymbol{\theta}) = p(T_{t,1}{=}\varepsilon_1, .., T_{t,K}{=}\varepsilon_K|\boldsymbol{A};\boldsymbol{\theta})$ and $\boldsymbol{\zeta}_t(\boldsymbol{\varepsilon},\boldsymbol{\varsigma}) = p(\boldsymbol{T}_t{=}\boldsymbol{\varepsilon}, \boldsymbol{T}_{t+1}{=}\boldsymbol{\varsigma}|\boldsymbol{A};\boldsymbol{\theta})$, defined over trait vectors $\boldsymbol{\varepsilon}$ and $\boldsymbol{\varsigma}$ in $\{0,1\}^K$. The marginal posterior probabilities for the cultural trait $\gamma_{r,t} = p(T_{t,r}|\boldsymbol{A};\boldsymbol{\theta})$ and marginal posterior probabilities of transition $\zeta_{r,t} = p(T_{t,r}, T_{r,t+1}|\boldsymbol{A};\boldsymbol{\theta})$ are then computed simply by marginalizing:

$$\begin{cases} \gamma_{r,t}(T) &= \sum\limits_{\boldsymbol{\varepsilon}\in\{0,1\}^K|\varepsilon(r)=T} \gamma_t(\boldsymbol{\varepsilon}) \\ \zeta_{r,t}(T,U) &= \sum\limits_{(\boldsymbol{\varepsilon},\boldsymbol{\varsigma})\in\{0,1\}^K\times\{0,1\}^K|\varepsilon(r)=T,\varsigma(r)=U} \zeta_t(\boldsymbol{\varepsilon},\boldsymbol{\varsigma}) \end{cases} \tag{1}$$

It should be noted that the inference problem is only tractable when the number of regions K remains low. Indeed the transition matrix has size 2^{2K}, which is already around 10^6 for $K = 10$ regions. If dt is sufficiently small, we can make the assumption that at most one transition can occur at each time step. Thus each multivariate state can only map onto $K + 1$ states (the state itself plus all the states corresponding to transition in one region), which means that the transition matrix has no more than $2^K(K + 1)$ non-zero values. We note C_t the transition from t to $t + 1$, with $C_t = r$ if the value of the trait in region r (and only region r) has changed between $t - 1$ and t, and $C_t = 0$ if no region has changed its state. The transition matrix then rewrites to:

$$p(C_t|\boldsymbol{T}_t) = \begin{cases} R_{\text{up}}(t,r)dt & \text{if } C_t > 0 \text{ and } T_{t,r} = 0 \\ R_{\text{down}}(t,r)dt & \text{if } C_t > 0 \text{ and } T_{t,r} = 1 \\ 1 - \sum_{r=1}^K p(C_t{=}r|\boldsymbol{T}_t) & \text{if } C_t = 0 \end{cases} \tag{2}$$

Then we note $\zeta_t(\boldsymbol{\varepsilon}, r)$ the posterior probability that $\boldsymbol{T}_t = \boldsymbol{\varepsilon}$ and that cultural trait in r has changed between t and $t + dt$ for $r \geq 1$, and $\zeta_t(\boldsymbol{\varepsilon}, 0)$ the posterior probability that $\boldsymbol{T}_{t+1} = \boldsymbol{T}_t = \boldsymbol{\varepsilon}$ (i.e. no trait change between t and $t + dt$).

A.2 M-step for binary traits

In the M-step we maximize $Q_{Ti}(\boldsymbol{\theta};\boldsymbol{\theta}^{old})$, $Q_{Te}(\boldsymbol{\theta}_{Te};\boldsymbol{\theta}^{old})$ and $Q_A(\boldsymbol{\theta}_A;\boldsymbol{\theta}^{old})$ with respect to new parameters $\boldsymbol{\theta}$:

$$\begin{cases} Q_{Ti}(\boldsymbol{\theta};\boldsymbol{\theta}^{old}) &= \sum_{r=1}^K (\gamma_{r,1}(0)\log(1-x_0) + \gamma_{r,1}(1)\log(x_0)) \\ Q_{Te}(\boldsymbol{\theta}_{Te};\boldsymbol{\theta}^{old}) &= \sum_{t=1}^{N-1} \sum_{\boldsymbol{\varepsilon}\in\{0,1\}^K} \sum_{\boldsymbol{\varsigma}\in\{0,1\}^K} \zeta_t(\boldsymbol{\varepsilon},\boldsymbol{\varsigma}) \log p(\boldsymbol{T}_{t+1} = \boldsymbol{\varsigma}|\boldsymbol{T}_t = \boldsymbol{\varepsilon}, \boldsymbol{\theta}_T) \\ Q_A(\boldsymbol{\theta}_A;\boldsymbol{\theta}^{old}) &= \sum_{\boldsymbol{T}} p(\boldsymbol{T}|\boldsymbol{\theta}^{old},\boldsymbol{A}) \log p(\boldsymbol{A}|\boldsymbol{T},\boldsymbol{\theta}_A) \\ &= \sum\limits_{t=1}^{N} \sum_{T\in\{0,1\}} \gamma_{t,r}(T) \log p(A_{t,r}|T_{t,r} = a, \boldsymbol{\theta}_A) \end{cases} \tag{3}$$

Maximizing $Q_{Ti}(\boldsymbol{\theta};\boldsymbol{\theta}^{old})$ yields $x_0 = \sum_r \gamma_{r,1}(1)/R$.

Maximizing $Q_{Te}(\boldsymbol{\theta}_{Te};\boldsymbol{\theta}^{old})$ with respect to trait evolution parameters $(b_{\text{up}}, b_{\text{down}}, \lambda, \xi)$ can be made use gradient methods. When dt is small enough that

multiple trait changes in one time step are negligible, $Q_{Te}(\boldsymbol{\theta}_{Te};\boldsymbol{\theta}^{old})$ rewrites as:

$$Q_{Te}(\boldsymbol{\theta}_{Te};\boldsymbol{\theta}^{old}) = \sum_{t=1}^{N-1} \sum_{\boldsymbol{\varepsilon}\in\{0,1\}^K} \sum_{C_t=0}^{K} \zeta_t(\boldsymbol{\varepsilon}, C_t) \log p(C_t|\boldsymbol{T}_t = \boldsymbol{\varepsilon}, \boldsymbol{\theta}_T) \tag{4}$$

The gradient and Hessian (necessary for the Laplace approximation) are obtained analytically by extracting first- and second-order derivatives from equations 4, 2 as well as equation 5 from the main manuscript.

Maximizing Q_A in equation 3 will depend on the particular form of the artifact production model. If each artifact can take a finite set of values $\boldsymbol{A}_{t,r} \in \{1, ..N_A\}$, then we can define parameters $p_A(A,T)$ as the probability that a given artifact takes value A if cultural trait is in state T. By noting $a(t,r,A)$ the counts of cultural artifacts of value A observed in region r at time step t, Q_A can be rewritten as:

$$Q_A(\boldsymbol{\theta}_A;\boldsymbol{\theta}^{old}) = \sum_{A=1}^{N_A} \sum_{T\in\{0,1\}} a(t,r,A) \log p_A(A,T) \sum_{r,t} \gamma_{t,r}(T) \tag{5}$$

Maximizing Q_A with the constraints $\sum_A p_A(A,T) = 1$ yields:

$$p_A(A,T) = \frac{\sum_{t,r} a(t,r,A)\gamma_{t,r}(T)}{\sum_{t,r,B} a(t,r,B)\gamma_{t,r}(T)} \tag{6}$$

A more general case will be that trait $T_{t,r}$ parametrizes as an exponential family distribution for artifact $T_{t,r}$. For example it will set the probability of observing one value if $T_{t,r}$ is binary (or can take a finite set of values), or it will determine the expected value of $T_{t,r}$ if $T_{t,r}$ can take continuous values or represents counts. Such framework allows to enter other covariates $F_{t,r}$ into the production model. In such cases maximizing Q_A to find the new values for $\boldsymbol{\theta}_A$ is equivalent to a GLM with observations $\{A_{t,r}\}$ and observations weights $\gamma_{t,r}$.

Appendix B Fitting continous ETC model

B.1 E-step for continuous traits

If traits are continuous and the production rule is itself linear-Gaussian, then the classical tools of linear dynamical systems can be used to infer parameters and the trajectory of cultural traits through time and space. Otherwise the inference problem is intractable, and a variety of approximation methods exist. Here we will restrict to Gaussian approximations since they allow analytical treatment, i.e. we will look for posterior distribution for cultural traits in the form:
$p(\boldsymbol{T}_t|\boldsymbol{A}_{(1,.,n)},\boldsymbol{\theta}) \approx q(\boldsymbol{T}) = \mathcal{N}(\boldsymbol{T};\boldsymbol{m},\boldsymbol{V})$. In particular we will be interested in the marginalized distribution $q(\boldsymbol{T}_t) = \mathcal{N}(\boldsymbol{T}_t,\boldsymbol{m}_t,\boldsymbol{V}_t)$ and the covariance between $\boldsymbol{T}_t$ and $\boldsymbol{T}_{t-1}$ noted $\boldsymbol{V}_{t,t-1}$.

We describe three different methods that look for gaussian approximations to the posterior: a moment method (or forward-backward algorithm)adapted from [3], and two variants derived from the Gaussian Processes (GP) formalism, the Laplace approximation and Expectation Propagation.

The forward backward method is largely adapted from [3, 4]. The E-step consists of three successive algorithms: a nonlinear recursive filter to compute $p(\boldsymbol{T}_t|\boldsymbol{A}_{(1,.,t)},\boldsymbol{\theta})$, which is where the Gaussian approximation is introduced; a Fixed Interval Smoothing algorithm, and a State-Space Covariance algorithm. The details of these algorithms are provided in Appendix B.4.

We can also define the E-step as a GP inference problem, where we again look for a gaussian approximation to the cultural trait posterior. While the Forward-Backward algorithm models only works on the distribution of traits on successive time steps, GP inference approximates the whole history of cultural traits as a multivariate normal distribution. We define a gaussian prior for $\boldsymbol{T}$, $p(\boldsymbol{T}|\boldsymbol{\theta}_T) = \mathcal{N}(\boldsymbol{\mu}, \boldsymbol{K})$, which is provided by the generative model (see below), and inference is run by looking at an approximate posterior $p(\boldsymbol{T}|\boldsymbol{\theta}_T, \boldsymbol{A}) \approx q(\boldsymbol{T}) = \mathcal{N}(\boldsymbol{m}, \boldsymbol{V})$ [5]. One possible concern with the moment method is that errors may accumulate across time steps while we run gaussian approximations separately at each time step. By looking at an gaussian approximation directly over the entire dataset, we may significantly reduce this error. This is however at the expense of higher computations demands: while the moment algorithm runs linearly in time (i.e. $O(n)$), the different GP algorithm will in general run cubically in the number of observations, and thus of time (i.e. $O(n^3)$. This constraint can be alleviated in two directions: first, instead of using all of the time points in the dataset, we can restrict to time point with corresponding observations (artifacts). Second we may use sparse approximations to GP inference, which provide much more scalable solutions [5]. We have not however explored this second possibility in this work.

From the auto-regressive model for cultural trait represented by equation 9 of main manuscript, it follows that the prior over $\boldsymbol{T}$ will be normally distributed. The prior mean and covariances can be evaluated iteratively:

$$\boldsymbol{\mu}_t = \boldsymbol{M}\boldsymbol{\mu}_{t-1} + \boldsymbol{J}_t \tag{7}$$

with $\boldsymbol{\mu}_0 = x_0\mathbf{1}$.

The prior covariance for $\boldsymbol{T}_t$ follows:

$$\boldsymbol{K}_{t,t} = \left(\sigma^2 dt \sum_{v=0}^{t-1} (\boldsymbol{M}^T\boldsymbol{M})^v + \sigma_0^2 (\boldsymbol{M}^T\boldsymbol{M})^t\right) \tag{8}$$

The covariance between traits at two different time steps t and $t+u$ $(0 \leq u \leq n-t)$ is given by:

$$\boldsymbol{K}_{t,t+u} = \boldsymbol{M}^{u-1}\boldsymbol{K}_{t,t} = \boldsymbol{M}^{u-1}\left(\sigma^2 dt \sum_{v=0}^{t-1} (\boldsymbol{M}^T\boldsymbol{M})^v + \sigma_0^2 \boldsymbol{I}(\boldsymbol{M}^T\boldsymbol{M})^t\right) \tag{9}$$

If the connectivity matrix $\boldsymbol{G}$ is symmetric, then $\boldsymbol{M}$ will be as well and $(\boldsymbol{M}^T\boldsymbol{M})^t = \boldsymbol{M}^{2t}$. Once the mean and covariance for $\boldsymbol{T}$ are built, we select the sub-vector $\boldsymbol{m}_{obs}$ and sub-matrix $\boldsymbol{K}_{obs}$ corresponding to data points $\boldsymbol{T}_{obs}$ with at least one observation (artifact). Then the GP inference corresponds to finding an approximate posterior $\mathcal{N}(\boldsymbol{T}_{obs}; \boldsymbol{m}_{obs}, \boldsymbol{V}_{obs})$ for $\boldsymbol{T}_{obs}$, given a prior $p(\boldsymbol{T}_{obs}) = \mathcal{N}(\boldsymbol{T}_{obs}; \boldsymbol{\mu}_{obs}, \boldsymbol{K}_{obs})$ and a series of observations $\{A_{tr}\}$ that each depends only on the value of the trait at one data point. The GP framework is extremely flexible as to the type of observations: they can be binary (GP classification), continuous (GP regression), or as for GLMs be parametrized by any exponential-family distribution, such as Poisson distribution for count data. The GPML toolbox for Matlab [6] allows to run inference for all these cases using a variety of different algorithms that each represent one case of gaussian approximation to the posterior. Here we use two different algorithms, Laplace approximation and Expectation Propagation (EP). Laplace approximation consists of identifying the mode $\boldsymbol{m}$ of the posterior distribution, i.e. the maximum a posteriori (MAP) value for the traits $arg\ \max_{\boldsymbol{T}}(p(\boldsymbol{T}|\boldsymbol{\theta}_T)p(\boldsymbol{A}|\boldsymbol{T}))$. The posterior covariance matrix is given by the Hessian of the log-posterior function evaluated at $\boldsymbol{m}$. EP is another approximation which usually provides more accurate approximation than Laplace for binary observations [7]. See [5] for a detailed presentation of these algorithms.

The GP inference algorithm outputs the posterior for traits corresponding to observed data points $q(\boldsymbol{T}_{obs}) = \mathcal{N}(\boldsymbol{m}_{obs}, \boldsymbol{V}_{obs})$. To recover the full posterior for all points in the data set, we used the prediction equations:

$$\boldsymbol{m}_t = \boldsymbol{\mu}_t + \boldsymbol{K}_{t,obs}\boldsymbol{K}_{obs}^{-1}(\boldsymbol{m}_{obs} - \boldsymbol{\mu}_{obs}) \tag{10}$$

$$\boldsymbol{V}_t = \boldsymbol{K}_{t,t} - \boldsymbol{K}_{t,obs}^T\boldsymbol{V}_{obs}^{-1}\boldsymbol{K}_{t,obs} \tag{11}$$

$$\boldsymbol{V}_{t-1,t} = \boldsymbol{K}_{t-1,t} - \boldsymbol{K}_{t-1,obs}^T\boldsymbol{V}_{obs}^{-1}\boldsymbol{K}_{t,obs} \tag{12}$$

where $\boldsymbol{K}_{t,obs}$ is the submatrix from $\boldsymbol{K}$ representing the prior covariance between traits at time point t and traits at all observed data points.

Once the gaussian approximation have been computed, using any of the methods presented above, we will interested in computing second moments of $\boldsymbol{T}_t$, and of $\boldsymbol{T}_t$ and $\boldsymbol{T}_{t-1}$ jointly. We have directly:

$$\boldsymbol{W}_t = <\boldsymbol{T}_t\boldsymbol{T}_t^T>_{q(\boldsymbol{T}_t)} = \boldsymbol{V}_t + \boldsymbol{m}_t\boldsymbol{m}_t^T \tag{13}$$

$$\boldsymbol{W}_{t-1,t} = <\boldsymbol{T}_t\boldsymbol{T}_{t-1}^T>_{q(\boldsymbol{T}_t,\boldsymbol{T}_{t-1})} = \boldsymbol{V}_{t-1,t} + \boldsymbol{m}_{t-1}\boldsymbol{m}_t^T \tag{14}$$

B.2 M-step for continuous traits

In the M-step we maximize $Q_{Ti}(\boldsymbol{\theta};\boldsymbol{\theta}^{old})$, $Q_{Te}(\boldsymbol{\theta}_{Te};\boldsymbol{\theta}^{old})$ and $Q_A(\boldsymbol{\theta}_A;\boldsymbol{\theta}^{old})$ with respect to parameters $\boldsymbol{\theta}$.

$$\begin{array}{rcl} Q_{Ti}(\boldsymbol{\theta};\boldsymbol{\theta}^{old}) & = & \int q(\boldsymbol{T}_0)\log\mathcal{N}(\boldsymbol{T}_0; x_0, \sigma_0^2 I) \\ & = & \sum_r < -(x-x_0)^2/(2\sigma_0^2) - \log\sigma_0^2/2 >_{x\sim q_{0r}} + const \end{array} \tag{15}$$

(note that actually the sum is not restricted to time step 0 but also applies to all regions created *de novo* and their time of creation)

$$<(x-x_0)^2>_{x\sim q_{0r}} = <x^2>_{x\sim q_{0r}} - 2x_0<x^2>_{x\sim q_{0r}} + x_0^2 = W_0(r,r) - 2x_0\mu_0(r) + x_0^2 \tag{16}$$

Plugging equation 16 into equation 15 and maximizing over x_0 and σ_0^2, we obtain:

$$\left\{\begin{array}{rcl} x_0 & = & \sum_r \boldsymbol{\mu}_0(r)/R \\ \sigma_0^2 & = & tr(\boldsymbol{W}_0)/K - x_0^2 \end{array}\right. \tag{17}$$

tr is the trace operator: $tr(\boldsymbol{M}) = \sum_r M(r,r)$. The corresponding maximum value is $Q_{Ti}(\boldsymbol{\theta};\boldsymbol{\theta}^{old}) = R(1 - \log\sigma_0^2)/2$.

We now seek to maximize $Q_{Te}(\boldsymbol{\theta}_{Te};\boldsymbol{\theta}^{old})$. From equation 9 of the main manuscript, we have:

$$\begin{array}{rcl} \log p(\boldsymbol{T}_t|\boldsymbol{T}_t - 1, \boldsymbol{\theta}_{Te}) & = & \log\mathcal{N}(\boldsymbol{T}_t - \boldsymbol{M}\boldsymbol{T}_{t-1} - \boldsymbol{J}_t, \sigma^2 dt\boldsymbol{I}) \\ & = & -\frac{1}{2\sigma^2 dt}||\boldsymbol{T}_t - \boldsymbol{M}\boldsymbol{T}_{t-1} - \boldsymbol{J}_t||^2 - \frac{K}{2}\log(2\pi\sigma^2 dt) \end{array} \tag{18}$$

Plugging this into equation 11 of the main manuscript, we obtain:

$$\left\{\begin{array}{rcl} Q_{Te}(\boldsymbol{\theta}_{Te};\boldsymbol{\theta}^{old}) & = & -\frac{Kn}{2}\log(2\pi\sigma^2 dt) - \frac{1}{2\sigma^2 dt}Z \\ Z & = & \sum_{t=1}^n < \boldsymbol{T}_t^T\boldsymbol{T}_t + \boldsymbol{T}_{t-1}^T\boldsymbol{M}^T\boldsymbol{M}\boldsymbol{T}_{t-1} + \boldsymbol{J}_t^T\boldsymbol{J}_t \\ & & -2\boldsymbol{T}_t^T\boldsymbol{M}\boldsymbol{T}_{t-1} - 2\boldsymbol{T}_t\boldsymbol{J}_t^T + 2\boldsymbol{J}_t^T\boldsymbol{M}\boldsymbol{T}_{t-1} >_q \end{array}\right. \tag{19}$$

The sum in Z is defined in terms of first and second moments of the distributions $q(\boldsymbol{T}_t)$ and $q(\boldsymbol{T}_t, \boldsymbol{T}_{t-1})$

$$\begin{aligned} Z = \textstyle\sum_t tr(\boldsymbol{W}_t) + s(\sum_t \boldsymbol{W}_{t-1} \circ \boldsymbol{M}^T\boldsymbol{M}) \sum_t \boldsymbol{J}_t^T \boldsymbol{J}_t - 2s(\boldsymbol{M} \circ \sum_t \boldsymbol{W}_{t-1,t}) \\ -2\textstyle\sum_t \boldsymbol{\mu}_t \boldsymbol{J}_t + 2s(\boldsymbol{M}^T \circ \sum_t \boldsymbol{\mu}_{t-1}\boldsymbol{J}_t) \end{aligned} \tag{20}$$

$\circ$ is the Hadamard product, i.e. $(\boldsymbol{M} \circ \boldsymbol{N})_{ij} = M_{ij}N_{ij}$, and s refers to the sum of all elements in a matrix ($s(\boldsymbol{M}) = \sum_{ij} M_{ij}$). The range of subscript t in this equation and the followings is from 1 to n.

By substiting the definitions of $\boldsymbol{M}$ and $\boldsymbol{J}_t$ (equations 9 of the main manuscript) into equation 20, we find that Z is a bilinear function of the subset of parameters $\boldsymbol{\nu} = (\gamma, \lambda, \xi, \rho)$, that is:

$$\begin{cases} Z &= dt^2\, \boldsymbol{\nu}^T \boldsymbol{C} \boldsymbol{\nu} - 2dt\, \boldsymbol{d}^T \boldsymbol{\nu} + const, \text{ with} \\ \boldsymbol{C} &= \begin{bmatrix} Kn & \mathbf{1}^T \sum \boldsymbol{E}_t & \mathbf{1}^T \tilde{\boldsymbol{G}} \sum \boldsymbol{\mu}_{t-1} & -\mathbf{1}^T \sum \boldsymbol{\mu}_{t-1} \\ \mathbf{1}^T \sum \boldsymbol{E}_t & \sum \boldsymbol{E}_t^T \boldsymbol{E}_t & s(\tilde{\boldsymbol{G}} \circ \sum \boldsymbol{\mu}_{t-1} \boldsymbol{E}_t^T) & -\sum \boldsymbol{\mu}_{t-1}^T \boldsymbol{E}_t \\ \mathbf{1}^T \tilde{\boldsymbol{G}} \sum \boldsymbol{\mu}_{t-1} & s(\tilde{\boldsymbol{G}} \circ \sum \boldsymbol{\mu}_{t-1} \boldsymbol{E}_t^T) & \beta_2 & -\beta_1 \\ -\mathbf{1}^T \sum \boldsymbol{\mu}_{t-1} & -\sum \boldsymbol{\mu}_{t-1}^T \boldsymbol{E}_t & -\beta_1 & \beta_0 \end{bmatrix} \\ \boldsymbol{d} &= \begin{bmatrix} \mathbf{1}^T(\boldsymbol{\mu}_n - \boldsymbol{\mu}_0) \\ \sum (\boldsymbol{\mu}_t - \boldsymbol{\mu}_{t-1})^T \boldsymbol{E}_t \\ s(\tilde{\boldsymbol{G}} \circ \sum \boldsymbol{W}_{t-1,t}) - \beta_1 \\ \beta_0 - tr(\sum \boldsymbol{W}_{t-1,t}) \end{bmatrix} \end{cases} \tag{21}$$

We have defined $\boldsymbol{\chi} = \sum_t \boldsymbol{W}_{t-1}$, $\beta_0 = tr(\boldsymbol{\chi})$, $\beta_1 = s(\boldsymbol{\chi} \circ \tilde{\boldsymbol{G}})$ and $\beta_2 = s(\boldsymbol{\chi} \circ \tilde{\boldsymbol{G}}^2)$. The subindex t has been omitted from the sums. When some of the parameters in $\boldsymbol{\nu}$ are fixed (i.e. ξ if we look at models without cultural diffusion), we simply remove rows and columns corresponding to the fixed parameters in $\boldsymbol{C}$ and $\boldsymbol{d}$.

Maximizing $Q_{Te}(\boldsymbol{\theta}_{Te}; \boldsymbol{\theta}^{old})$ w.r.t. $\boldsymbol{\nu}$ is equivalent to minimizing Z, which is achieved at

$$\boldsymbol{\nu} = \frac{1}{dt} \boldsymbol{C}^{-1} \boldsymbol{d} \tag{22}$$

In order to find the new value of σ^2, we look for the maximum of $Q_{Te}(\boldsymbol{\theta}_{Te}; \boldsymbol{\theta}^{old})$ w.r.t. σ^2 using equation 19, yielding:

$$\sigma^2 = \frac{Z}{Kndt} \tag{23}$$

where Z is computed using the values of $\boldsymbol{M}$ and $\boldsymbol{J}_t$ with the new values of ρ, ξ, λ and γ.

In summary we can use equations 17,22, 23 to update the values of parameters $\boldsymbol{\theta}_T$. In case the artifact production model also contains some parameters $\boldsymbol{\theta}_A$, we will seek to maximize:

$$Q_A(\boldsymbol{\theta}_A) = \sum_{t,r} \int \mathcal{N}(T_{tr}; m_t(r), V_t(r,r)) \log p(\boldsymbol{A}_{tr} | T_{tr}, \boldsymbol{\theta}_A) dT_{tr} \tag{24}$$

In the case of count observations, where the observations are generated from Poisson distribution where the rate is defined as $\lambda(r,t) = \exp(\lambda_0 + \lambda_1 T_{t,r})$, we can use Newton's method to find best fitting values for parameters (λ_0, λ_1) as described in [3]. For the general case the integrals and their derivatives w.r.t parameters $\boldsymbol{\theta}_A$ have no analytical forms, but various approximations schemes can be applied, i.e. using Gaussian-Hermite quadrature [8]. Such approximations can be used to find new set of parameters $\boldsymbol{\theta}_A$ that maximizes Q_A.

B.3 Hessian

Confidence intervals and marginalized evidence both rely on computing the Hessian of the log-likelihood, which is related to the Hessian of $Q(\boldsymbol{\theta})$ through equation 14 of the main manuscript. Using equations 15, 16,19 and 21, we find that the Hessian of Q_T has the following form:

$$H_{Q_T} = \nabla\nabla Q_T(\boldsymbol{\theta}_T) = \begin{bmatrix} -dt\boldsymbol{C}/\sigma^2 & \mathbf{0} & \mathbf{0} & \mathbf{0} \\ \mathbf{0} & -\frac{Kn}{2\sigma^4} & 0 & 0 \\ \mathbf{0} & 0 & -K/\sigma_0^2 & 0 \\ \mathbf{0} & 0 & 0 & -\frac{K}{2\sigma_0^4} \end{bmatrix} \tag{25}$$

B.4 Forward backward method

These equations are directly derived when adapting the formalism of [3, 4] to the generative model of continouous ETC.

B.4.1 Nonlinear recursive filter

We define the approximations $p(\boldsymbol{T}_t|\boldsymbol{A}) = \mathcal{N}(\boldsymbol{x}_{t|t}, \boldsymbol{W}_{t|t})$ and $p(\boldsymbol{T}_{t+1}|\boldsymbol{A}_{(1,.,t)}) = \mathcal{N}(\boldsymbol{x}_{t+1|t}, \boldsymbol{W}_{t+1|t})$, where the distributions of traits are conditioned on all artifacts up to time step t. Mean and covariance matrices are initiated at the first time step by $\boldsymbol{x}_{0|0} = x_0$ and $W_{0|0} = \sigma_0^2\boldsymbol{I}$. From equation 9 of the main manuscript, we have the following prediction rule:

$$\boldsymbol{x}_{t|t-1} = \boldsymbol{M}\boldsymbol{x}_{t-1|t-1} + \boldsymbol{J}_t \tag{26}$$

$$\boldsymbol{W}_{t|t-1} = \boldsymbol{M}\boldsymbol{W}_{t-1|t-1}\boldsymbol{M} + \sigma^2 dt\boldsymbol{I} \tag{27}$$

In the special case where a new region r is initiated at t with parent region s (i.e. split or a political state in two), the value of T_t is inherited from the parent region, i.e. $x_{t|t}(r) = x_{t|t}(s)$ and $W_{t|t}(r,r) = W_{t|t}(r,s) = W_{t|t}(s,r) = W_{t|t}(s,s)$. For creations *de novo*, we have $x_{t|t}(r) = x_0$ and $W_{t|t}(r,r) = \sigma_0^2$.

In the case of continuous artifacts generated from gaussian noise with variance ϵ^2, i.e. gaussian observations $A_{tr} \sim \mathcal{N}(T_{tr}, \epsilon^2)$), the posterior distribution after integrating new observation $\boldsymbol{A}_t$ is itself gaussian, and we can calculate the new distribution $p(\boldsymbol{T}_t|A_{1..t}) = \mathcal{N}(\boldsymbol{T}_t; \boldsymbol{x}_{t|t}, \boldsymbol{W}_{t|t})$ by following the classical linear dynamical system:

$$\boldsymbol{x}_{t|t} = \boldsymbol{x}_{t|t-1} + \boldsymbol{K}_t(\boldsymbol{A}_t - \boldsymbol{x}_{t|t-1}) \tag{28}$$

$$\boldsymbol{W}_{t|t} = (I - \boldsymbol{K}_t)\boldsymbol{W}_{t|t-1} \tag{29}$$

where we have used the Kalman gain matrix $\boldsymbol{K}_t = \boldsymbol{W}_{t|t-1}(\boldsymbol{W}_{t|t-1} + \epsilon^2 I)^{-1}$.

If observations are not gaussian, the step is not exact and we look for Gaussian approximations $p(\boldsymbol{T}_t|A_{1..t}) = \mathcal{N}(\boldsymbol{T}_t; \boldsymbol{x}_{t|t}, \boldsymbol{W}_{t|t})$. If artifacts take binary values, the distribution set by:

$$\log p(\boldsymbol{T}_t|H_t) \propto (\boldsymbol{T}_t - \boldsymbol{x}_{t|t-1})^T \boldsymbol{W}_{t-1|t-1}^{-1}(\boldsymbol{T}_t - \boldsymbol{x}_{t|t-1}) + \sum_r (A_{tr}^+ \log\sigma(T_{tr}) + A_{tr}^- \log\sigma(-T_{tr})) \tag{30}$$

We recognize the mode $\boldsymbol{x}_{t|t}$ as being the solution of a logistic regression problem with observations $\boldsymbol{A}_t$, identity design matrix $\boldsymbol{I}$ and gaussian prior over weights $p(\boldsymbol{x}) = \mathcal{N}(\boldsymbol{x}; \boldsymbol{x}_{t|t-1}, \boldsymbol{W}_{t|t-1})$ (this can also be viewed as a Gaussian Process classification problem with prior mean $\boldsymbol{x}_{t|t-1}$ and prior covariance $\boldsymbol{W}_{t|t-1}$). The mode

can simply be found solving this logistic regression problem, e.g using the IRLS algorithm. $\boldsymbol{W}_{t|t}$ is the inverse of the Hessian of $\log p(\boldsymbol{T}_t|H_t)$ evaluated at the mode, i.e. it is the inverse of the observed Fisher information matrix.

In general, if observation process (artifacts) are derived from an generalized family distribution, i.e. Poisson counts, we can find the mode $\boldsymbol{x}_{t|t}$ and $\boldsymbol{W}_{t|t}$ by solving a GLM with prior mean gaussian prior over weights $p(\boldsymbol{x}) = \mathcal{N}(\boldsymbol{x}; \boldsymbol{x}_{t|t-1}, \boldsymbol{W}_{t|t-1})$). The solution is equivalent to that found by the recursive method proposed by [3].

B.4.2 Fixed Interval Smoothing

Defining $\boldsymbol{B}_t = \boldsymbol{W}_{t|t}\boldsymbol{M}\boldsymbol{W}_{t+1|t}^{-1}$ [9], we have the backward recursive equations:

$$\boldsymbol{m}_t = \boldsymbol{x}_{t|t} + \boldsymbol{B}_t(\boldsymbol{m}_{t+1} - \boldsymbol{x}_{t+1|t}) \tag{31}$$

$$\boldsymbol{V}_t = \boldsymbol{W}_{t|t} + \boldsymbol{B}_t(\boldsymbol{V}_{t+1} - \boldsymbol{W}_{t+1|t})\boldsymbol{B}_t^T \tag{32}$$

$$\boldsymbol{V}_{t,t+1} = \boldsymbol{B}_t \boldsymbol{V}_{t+1} \tag{33}$$

(this last equation from [10])

B.5 Relative influence of vertical transmission, horizontal transmission and ecological factors

We show here how the relative influence of vertical transmission I_v, horizontal transmission I_h and ecology I_e onto the evolution of the cultural trait can be estimated from a dataset. Once maximum likelihood ETC parameters have been identified, we estimate the proportion of the variance of T that can be explained by each factor:

$$\begin{cases} I_v &= V_v/SV \\ I_h &= V_h/SV \\ I_e &= V_e/SV \end{cases} \tag{34}$$

where $SV = V_v + V_h + V_e + V_n$ is the sum of variance, V_v, V_h, V_e and V_n are the variance terms of T due to vertical transmission, horizontal transmission, ecology and noise respectively. The different terms can be computed analytically by looking how the cultural trait in one region $T(r,t)$, in different regions $T(s,t)$ and the ecological factor $E(r,t)$ determine cultural trait in the same region the cultural trait after one generation $T(r, t+T_g)$, where T_g is the duration of a generation (typically ~ 25 years). For the case of continuous traits, we decompose the dynamics of $\boldsymbol{T}$ from equation 9 of the main manuscript into $\boldsymbol{T} = \boldsymbol{T}_v + \boldsymbol{T}_h + \boldsymbol{T}_e + \boldsymbol{T}_n$, where $\boldsymbol{T}_h = \tilde{\boldsymbol{T}}_h - \boldsymbol{T}_v$, and the dynamics of the different components follow the different equations:

$$\begin{cases} \frac{d\boldsymbol{T}_v}{dt} &= -\rho \boldsymbol{T}_v + \gamma \boldsymbol{1} \\ \frac{d\tilde{\boldsymbol{T}}_h}{dt} &= -\boldsymbol{M}\tilde{\boldsymbol{T}}_h + \gamma \boldsymbol{1} \\ \frac{d\boldsymbol{T}_e}{dt} &= -\rho \boldsymbol{T}_v + \boldsymbol{E}(t) \\ \frac{d\boldsymbol{T}_n}{dt} &= -\boldsymbol{M}\boldsymbol{T}_n + \boldsymbol{\eta}(t) \end{cases} \tag{35}$$

with initial conditions $\boldsymbol{T}_v(t_0) = \tilde{\boldsymbol{T}}_h(t_0) = \boldsymbol{T}(t_0)$ and $\boldsymbol{T}_e(t_0) = \boldsymbol{T}_n(t_0) = 0$. The solution to first (deterministic) differential equations are:

$$\begin{cases} \boldsymbol{T}_v(t) &= \boldsymbol{T}(t_0) + e^{-\rho(t-t_0)}(\gamma \boldsymbol{1} - \boldsymbol{T}(t_0)) \\ \tilde{\boldsymbol{T}}_h(t) &= \boldsymbol{T}(t_0) + e^{-\boldsymbol{M}(t-t_0)}(\gamma \boldsymbol{1} - \boldsymbol{T}(t_0)) \\ \boldsymbol{T}_e(t) &= (\boldsymbol{I} - e^{-\boldsymbol{M}(t-t_0)})\boldsymbol{E}(t_0) \end{cases} \tag{36}$$

Here we assumed that the ecological factor is nearly constant within the time range of one generation. We use these equations to compute the value of $\boldsymbol{T}_v, \boldsymbol{T}_h$ and $\boldsymbol{T}_e$ after one generation (at $t = t_0 + T_g$) for all starting points t_0 in the dataset. Variance terms V_v, V_h and V_e are obtained by computing the variance of the respective terms over regions and time points. Finally, we recognized that $\boldsymbol{T}_n$ is a multivariate Orstein-Uhlenbeck process, whose variance is $\mathrm{Var}(\boldsymbol{T}_n(t)) = \sigma^2 \boldsymbol{M}^{-1}(\boldsymbol{I} - e^{-\boldsymbol{M}(t-t_0)})$.

B.6 Pseudo-Variance Inflation Factor

The discretized update equation for continuous trait (Equation 7 of the main manuscript) be rewritten as:

$$\Delta T_{t,r} = \gamma \times 1 + \rho \times (-T_{t-1,r}) + \lambda E_{t,r} + \xi U_{t,r} + \sigma \frac{1}{\sqrt{dt}} \eta \tag{37}$$

with $\Delta T_{t,r} = T_{t,r} - T_{t-1,r}$ and $\boldsymbol{U}_t = \tilde{\boldsymbol{G}} \boldsymbol{T}_t$. We recognize here the generative model of multiple linear regression where $\Delta T_{t,r}$ is the dependent variable, $\boldsymbol{x}_{t,r} = (1, -T_{t-1,r}, E_{t,r}, U_{t,r})$ are the regressors and $(\gamma, \rho, \lambda, \xi)$ are their corresponding weights. If the exact values of the traits are provided (e.g. the cultural traits $\boldsymbol{T}_t$ are observed and not latent variables), the maximum-likelihood estimates for the parameters can be derived using the standard solution for linear regression:

$$(\gamma, \rho, \lambda, \xi) = (X^T X)^{-1} (X \Delta \boldsymbol{T}) \tag{38}$$

where the KT rows of the matrix X and of the vector $\Delta \boldsymbol{T}$ are obtained by concatenating the values of $\boldsymbol{x}_{t,r}$ and $\Delta T_{t,r}$ over both regions and time steps.

Issues of near-colinearity of the regressors can be investigated using the Variance Inflation Factor or VIF. A VIF value is obtained for each column of X and indicates the identifiability of the corresponding parameter: a value close to 1 indicates good identifiability, while very high values indicate near-colinearity of the regressors and consequently poor identifiability (Supp Fig. 5).

In the general case, the cultural trait is not observed but inferred so we cannot directly use equation 38 or compute the VIF. Rather, we define the pseudo-VIF as the VIF where in the definition of the design matrix X we replace the (unknown) values of $\boldsymbol{T}_{t,r}$ by its expected value under the posterior $< T_{t,r} >_{\boldsymbol{T}|\boldsymbol{E},\boldsymbol{A}} = m_{t,r}$. A large pseudo-VIF indicates poor identifiability of the corresponding parameter. The converse is unfortunately not true: a low pseudo-VIF value is reassuring but does not guarantee good identifiability as issues may also arise due to the uncertainty related to the value of cultural traits. A parameter recovery analysis is the only way to guarantee good identifiability properties in general.

Appendix C Simulations

C.1 Binary cultural trait

We simulated an ETC model with $R = 6$ regions over a period of 1000 times steps. The duration of each time step was drawn from a uniform distribution between 0.1 and 0.2 arbitrary units of time. Region 1 was created from region 3 at time step 501. Region 3 was created *de novo* at time step 301. All other regions were created *de novo* at time step 1. Region 3 was destroyed at time step 600, while all other regions were maintained for the rest of the simulation. The graphical connectivity was generated as a Erdös-Rényi random graph, i.e. the connection between each pair of regions was drawn with a fixed probabilty $p = 0.3$ independently of other connections. The ecological factor E was generated from independent stochastic process in each region, obtained by

filtering a gaussian white noise process η_{rt}: $\tau_E E_{r,t} + E_{r,t-1} = 5.65 \sum_{u=1}^{\tau_E} \eta_{r,t-u}$. Parameters of the ETC were $b_{\text{up}} = b_{\text{down}} = .1$ (transition probabilities), $\lambda = .03$ (evoked parameter), $\xi = 0.03$ (diffusion parameter), $x_0 = 0$ (initial state of T).

We used binary artifacts with value 0 or 1. The number of artifacts per data point was drawn from a Poisson distribution with rate 0.4. For each artifact, the probability that the artifact takes value 1 was determined by the value of the trait at the same time and region: $p(A_{t,r} = 1|T_{t,r} = 1) = C_{\text{up}} = .6$, and $p(A_{t,r} = 1|T_{t,r} = 0) = C_{\text{down}} = 0$.

After times series for ecological factors, cultural traits and cultural artifacts were synthesized, we used the algorithm described in Appendix A to estimate ETC parameters $(x_0, b_{\text{up}}, b_{\text{down}}, \lambda, \xi, C_{\text{up}}, C_{\text{down}})$ from the observed data $(\boldsymbol{E}, \boldsymbol{A})$.

C.2 Continuous cultural trait

We simulated an ETC model with $R = 5$ regions over a period of 400 times steps, with fixed time step duration $dt = 0.05$ (for example representing a total span of 2000 years with time steps of 50 years). Region 2 was created from region 3 at time step 31. Region 1 was created *de novo* at time step 51. All other regions were created *de novo* at time step 1. Region 3 was destroyed at time step 300, while all other regions were maintained for the rest of the simulation. The graphical connectivity was generated as a Erdös-Rényi random graph, i.e. the connection between each pair of regions was drawn with a fixed probabilty $p = 0.91$ independently of other connections. The ecological factor E was generated from an independent stochastic process in each region, similarly to binary cultural trait simulations. Parameters of the ETC were $\rho = .08$ (cultural lability), $\lambda = 1$ (evoked parameter), $\xi = 0.1$ (diffusion parameter), $\gamma = .3$ (trait default value), $\sigma^2 = .2$ (noise variance), $x_0 = .6$ and $\sigma_0^2 = .5$ (mean and variance of initial state of T).

We used count variable for artifacts. The number of artifacts per data point was drawn from a Poisson distribution with rate 0.5. The value associated with each each artifact was sampled from a Poisson distribution with rate $\exp(T_{t,r})$.

After times series for ecological factors, cultural traits and cultural artifacts were synthesized, we used the algorithm described in section B to estimate ETC parameters $(x_0, \sigma_0^2, \rho, \lambda, \xi, \gamma, \sigma^2)$ from the observed data $(\boldsymbol{E}, \boldsymbol{A})$, using the three different approximation techniques for the E-step: moment method, Laplace approximation and Expectation-Propagation. The analysis was repeated for 100 different simulations of the model.

C.3 Two stage analysis

For the analysis comparing one-stage vs two-stage approaches, we simulated an ETC model with $R = 5$ regions over a period of 500 times steps, with fixed time step duration $dt = 0.05$. Region 2 was created from region 3 at time step 31. All other regions were created *de novo* at time step 1, and all regions were maintained until the end of the simulation. The graphical connectivity was generated as a Erdös-Rényi random graph with a fixed probability of connection $p = 0.91$. The ecological factor E was generated as in previous analyses, and we used the same parameters as for the continuous analysis above.

We used a continuous variable for artifacts. The number of artifacts per data point was drawn from a Poisson distribution whose rate changed over time: the rate was set to .1 for the first 400 time steps (scarcity of artifacts period) and jumped to 1 for the remaining 100 time steps (abundance of artifacts period). The value associated with each each artifact was sampled from a normal distribution with mean $T_{t,r}$ and variance $\epsilon^2 = 1$, so that the average of artifacts at any time point provides a direct unbiased estimate of the associated cultural trait.

After times series for ecological factors, cultural traits and cultural artifacts were synthesized, we compared the estimated provided by standard ETC method (with exact E step, since the observations are gaussian) with a two-stage procedure. In the two-step procedure, cultural traits are first estimated directly from cultural artifacts: estimated cultural trait $\hat{T}(r,t)$ is taken as the average of the value of all artifacts produced in region r in a window of 10 time steps centered on t. Then we estimate the ETC parameters by maximizing the likelihood over latent evolution $p(\hat{\boldsymbol{T}}|\boldsymbol{\theta}_T)$ and the likelihood over cultural production $p(\boldsymbol{A}|\hat{\boldsymbol{T}};\boldsymbol{\theta}_A)$. This is indeed equivalent to maximizing the log of the joint-likelihood $\log p(\boldsymbol{A};\hat{\boldsymbol{T}}|\boldsymbol{\theta}) = \log p(\boldsymbol{A}|\hat{\boldsymbol{T}},\boldsymbol{\theta}_A) + \log p(\hat{\boldsymbol{T}}|\boldsymbol{\theta}_T)$, which is equivalent to the M step of the E algorithm where the variance of posterior cultural traits is null. The analysis was repeated for 100 different simulations of the model.

C.4 Synchronous data analysis

We simulated data from an ETC model with continuous traits over 100 regions over 400 time steps ($dt = 0.02$). Regions were created according to a random phylogenetic tree, which was generated as follow. We started the tree from a single region at time step 1 (created de novo), at coordinates $(0,0)$ on a map. At each iteration, we randomly selected one region out of the *alive* regions. This region had a random number between 2 and 4 offspring regions, after a random duration following its creation. This region was *extinguished* at the moment it generated offspring regions. Ecological variables in each region were drawn from independent binary markov processes with a probability of $0.5dt$ of changing value at each time step. The location for each new region was taken at a distance Inferring individual-level processes from population-level patterns in cultural evolution. from the normal distribution both in the x and y direction. Non-diagonal elements in the connectivity matrix G scaled exponentially with the euclidian distance between each regions d_{rs}, i.e. $G_{rs} = e^{-d_{rs}}$. Parameters of the model were the following: $\rho = 0.5$, $\lambda = 2$, $\xi = 1$, $\sigma^2 = 0.002$, $\gamma = -1$, $x_0 = -0$. After simulating the data, we fitted the ETC model using synchronous data, i.e. the value of the cultural trait observed at the last time steps for all alive regions. We compared two variants of the ETC model: either the full model, or the model without horizontal transmission.

References

1. Rabiner L. Dune Patterns, Namib Desert, Namibia. vol. 77; 1989. Available from: http://ieeexplore.ieee.org/xpls/abs{_}all.jsp?isNumber=698{&}prod=JNL{&}arnumber=18626{&}arSt=257{&}ared=http://earthobservatory.nasa.gov/Newsroom/NewImages/images.php3?img{_}id=17050.

2. Bishop CM. Pattern Recognition and Machine Learning; 2006.

3. Smith AC, Brown EN. Estimating a State-Space Model from Point Process Observations. Neural Computation. 2003;15(5):965–991. doi:10.1162/089976603765202622.

4. Brown EN, Frank LM, Tang D, Quirk MC, Wilson MA. A statistical paradigm for neural spike train decoding applied to position prediction from ensemble firing patterns of rat hippocampal place cells. Journal of Neuroscience. 1998;18(18):7411–25.

5. Rasmussen CE, Williams CKI. Gaussian processes for machine learning.. vol. 14; 2004. Available from: http://www.gaussianprocess.org/gpml/chapters/RW.pdf.

6. Rasmussen CE, Nickisch H. Gaussian Processes for Machine Learning (GPML) Toolbox Carl. 2Journal of Machine Learning Research. 2010; p. 3011–30155. doi:10.1002/fee.1794.

7. Nickisch H, Rasmussen CE. Approximations for Binary Gaussian Process Classification. Journal of Machine Learning Research. 2008;9:2035–2078.

8. Liu Q, Pierce DA. A note on Gauss-Hermite quadrature. Biometrika. 1994;81(3):624–629. doi:10.1093/biomet/81.3.624.

9. Söderkvist I. An algorithm for Kalman filtering and smoothing with diagonal input covariance matrices. In: Computational techniques and applications, CTAC95: proceedings of the Seventh Biennial Conference. World Scientific; 1996. p. 875. Available from: http://www.diva-portal.org/smash/record.jsf?pid=diva2{%}3A1001365{&}dswid=-8412.

10. De Jong P, MacKinnon MJ. Covariances for Smoothed Estimates in State-space Models. Biometrika. 1988;75(3):601–602.
