# Supplementary figures and images for: Evoked and transmitted culture models: Using bayesian methods to infer the evolution of cultural traits in history
Source: PLoS One. 2022 Apr 7;17(4):e0264509. doi: 10.1371/journal.pone.0264509 (PMC8989295; doi:10.1371/journal.pone.0264509)

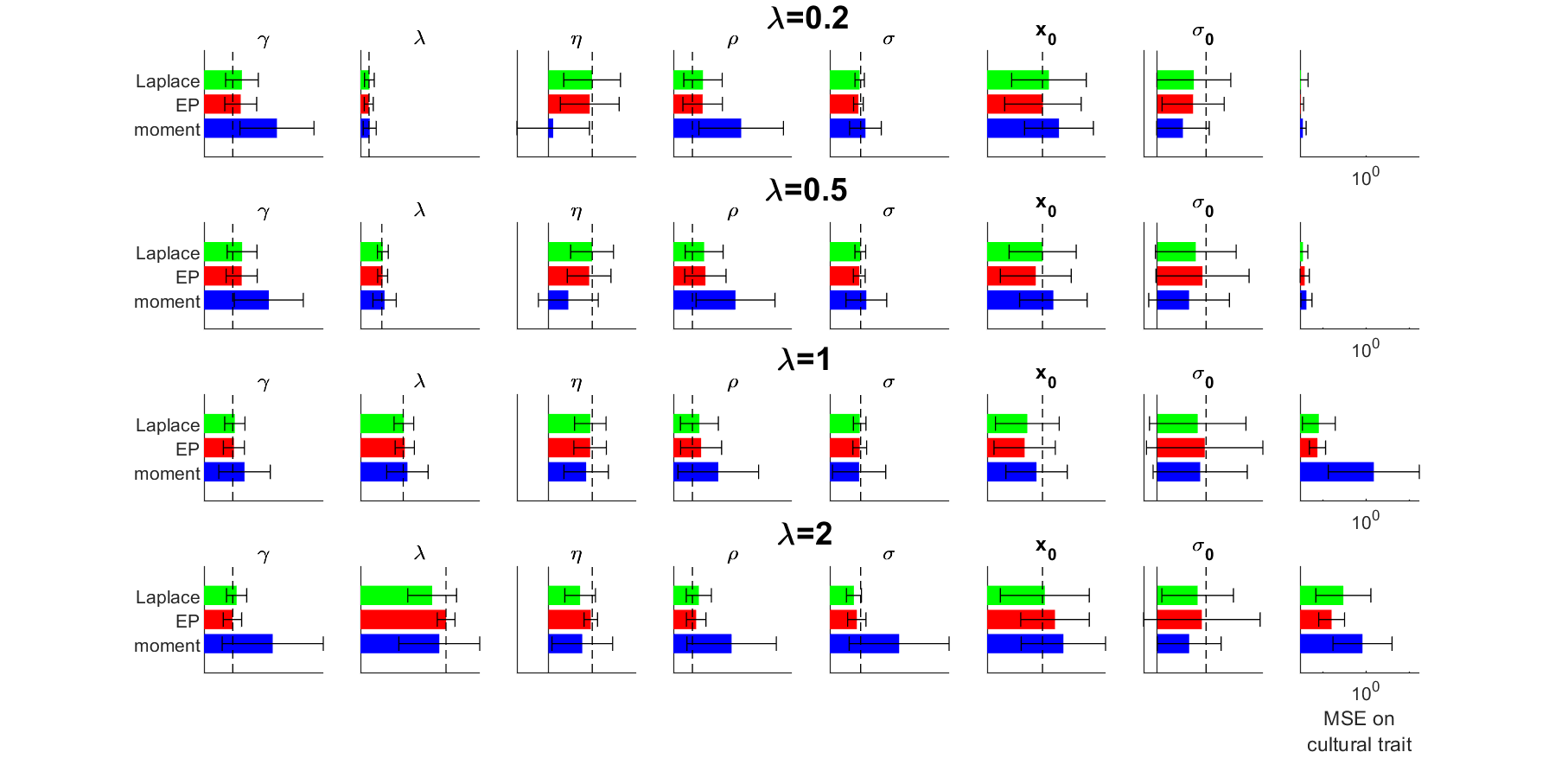

Supplement: S1 Fig — Each row represents a value of λ, and each column represents one parameter from the ETC model. The last column represents the MSE on the cultural trait. Legend as in Fig 4 of main manuscript. (PNG) [file pone.0264509.s002.png]

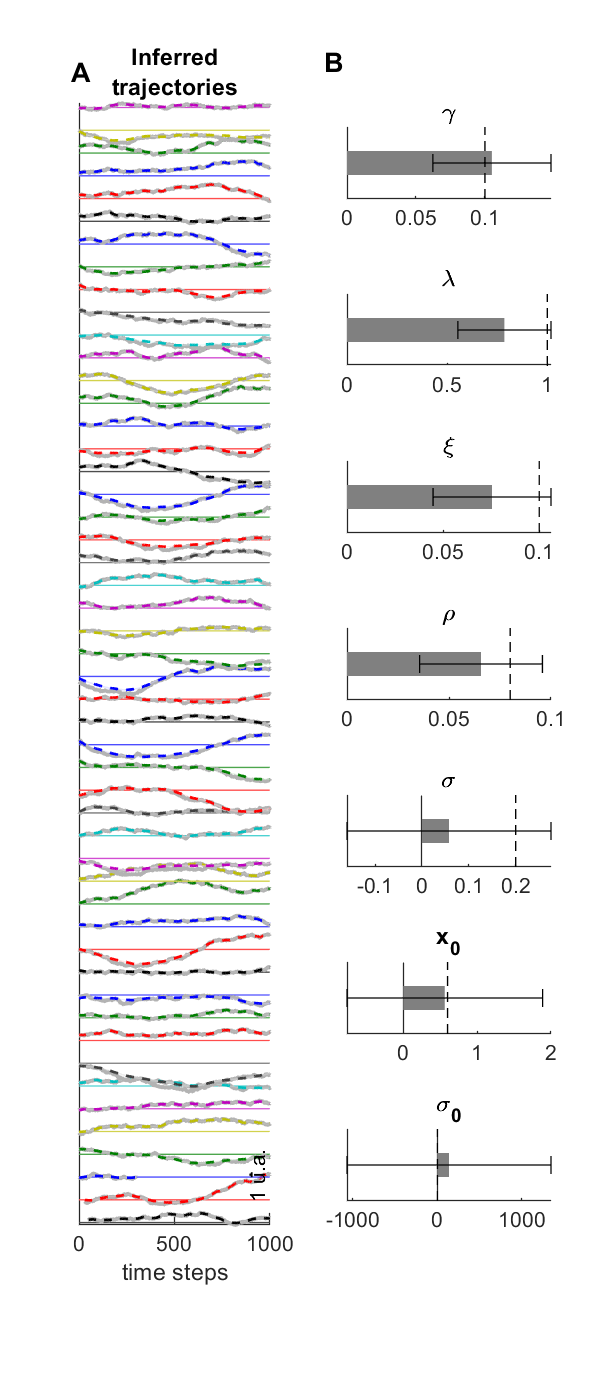

Supplement: S2 Fig — Panel A shows the true trajectories (full grey lines) and inferred trajectories using the moment method (dotted color lines) for each region. The overlap shows that all trajectories were well estimated. The parameters used for simulations are: γ = 0.1, λ = 1, ξ = 0.1, ρ = 0.08, σ = 0.2, x0 = 0.6, σ0 = 2. Panel B shows the estimated parameters for 100 simulations with same parameter values (bar: mean over simulations; error bar: standard deviation over simulations). Dotted lines indicate the true value of the parameter. (PNG) [file pone.0264509.s003.png]

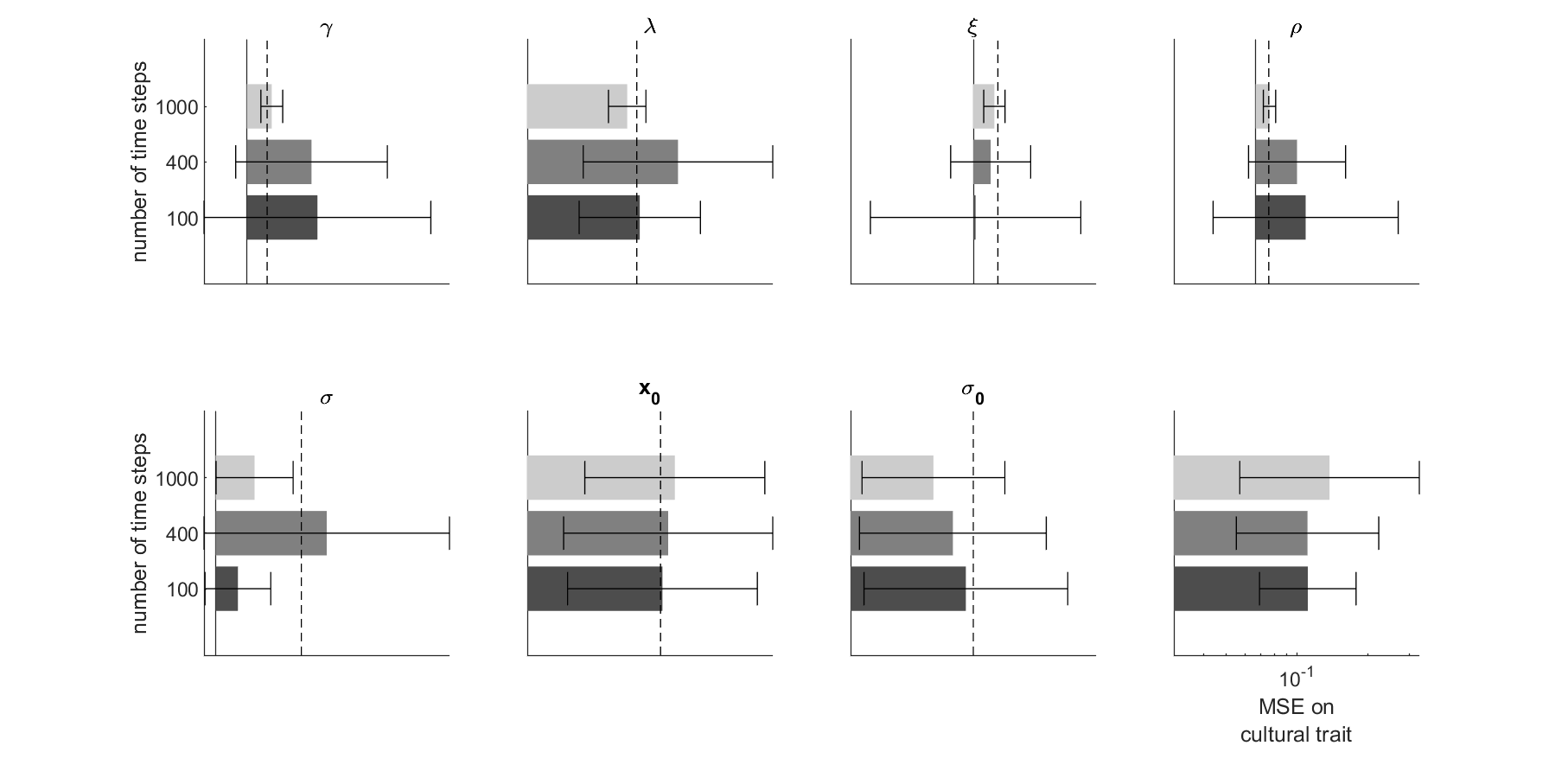

Supplement: S3 Fig — The parameters used for simulations are: γ = 0.1, λ = 1, ξ = 0.04, ρ = 0.08, σ = 0.2, x0 = 0.6, σ0 = 0.5. Each panel represents one parameter from the ETC model. Bars and error bars represent the mean and standard deviation of the estimated parameters over 100 simulations, respectively. The bottom right panel represents the MSE on the cultural trait. (PNG) [file pone.0264509.s004.png]

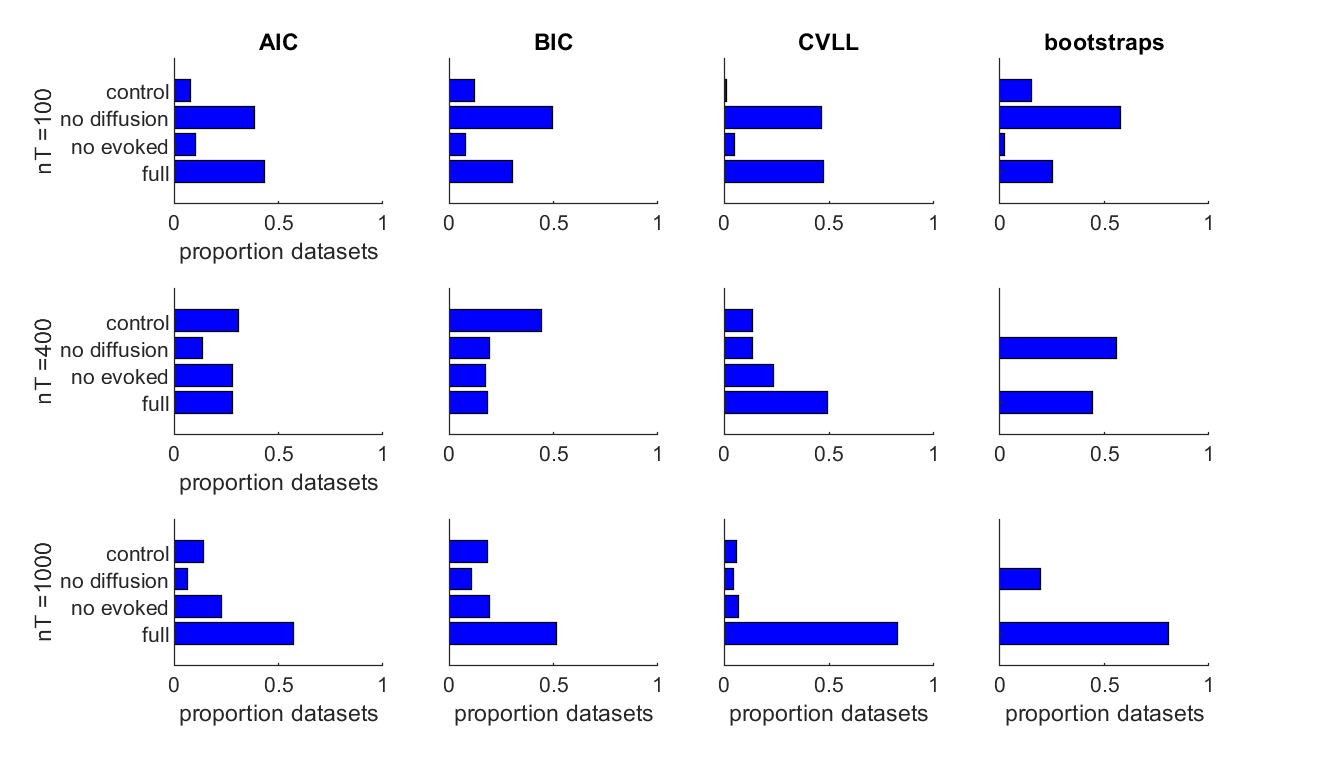

Supplement: S4 Fig — Each row corresponds to a different number of time steps T, each column corresponds to a different metric used for model comparison. The larger is the dataset, the more probable it is that the full model is correctly identified as the best model, for all types of metrics. (PNG) [file pone.0264509.s005.png]

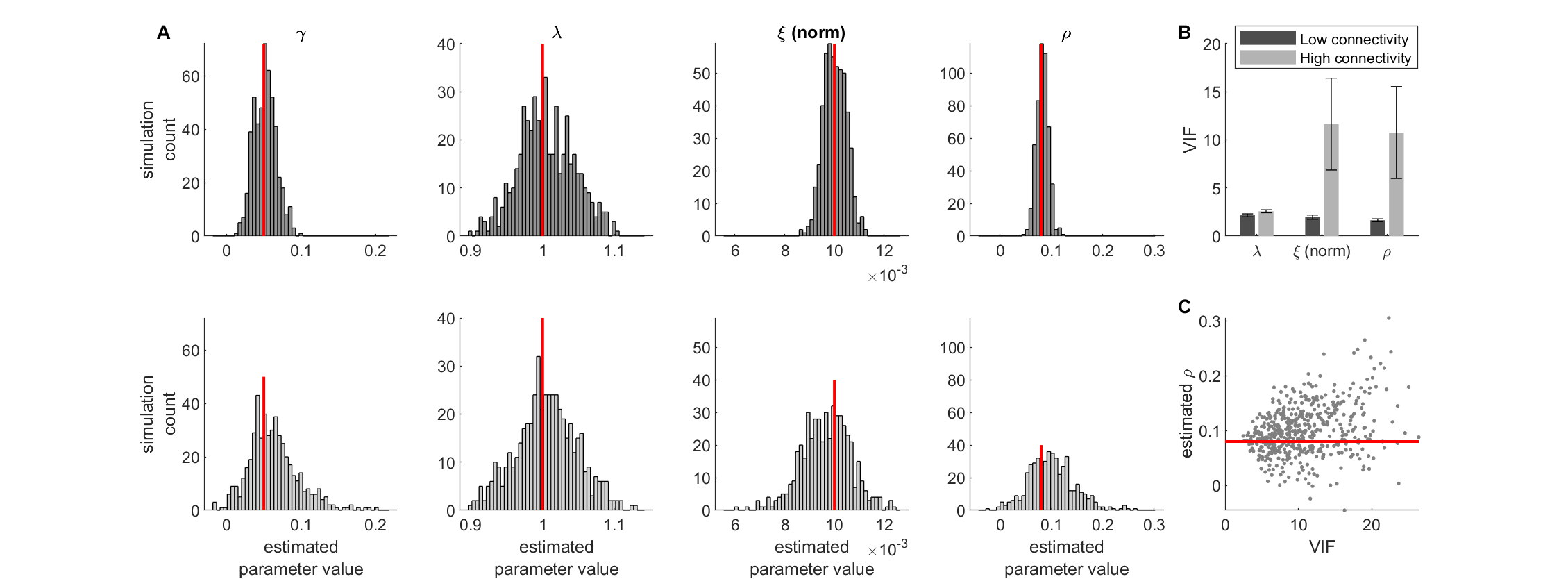

Supplement: S5 Fig — The probability of connectivity (with node strength Grs = 1) was set to pG = 0.5 in the high-connectivity model and pG = 0.05 in the low-connectivity model. In each simulation, an ETC model with K = 50 regions was simulated during 500 times steps, using the following value of parameters: γ = 0.05, λ = 1, ρ = 0.08, σ = 0.2, x0 = 0.6, σ0 = 0.1. The value of the diffusion parameter ξ was normalized to the overall probability of connection between each region pG (ξ = 0.01/pG) so that the overall influence of cultural diffusion was comparable between the high-connectivity and low-connectivity models. A. Distribution of parameters estimated from the models, from 500 simulations for both low-connectivity (top row) and high-connectivity (bottom row) matrices. The diffusion parameter is normalized to ξ/pG for better comparison. Red lines indicate the true value of the parameter. Note that the estimation of parameters γ, ξ and ρ are much less precise in the high connectivity network compared to the low-connectivity network, while the estimation of λ is mostly preserved. B. Variance Inflation Factor (VIF) for the regressors related to the λ, ξ and ρ parameters into the linear regression analysis, averaged over simulations (bar: mean; error bars: standard deviation), for low- and high-connectivity models. VIF values close to 1 indicate good identifiability of the parameters, while values above 5–10 indicate poor identifiability due to regressors being nearly colinear. Note the problem of colinearity in the high-connectiviy network affecting the identifiability of parametres ξ and ρ (but not λ). C. Relationship between VIF for regressor related to ρ and the estimated value of parameter ρ. Each dot represents a simulation. Red line indicates the true value. Larger estimation errors are obtained for simulations with large VIF. In other words, the relationship between VIF and estimation error is present within the same set of simulations. (PNG) [file pone.0264509.s006.png]
