# Supplementary material for: Evoked and transmitted culture models: Using bayesian methods to infer the evolution of cultural traits in history
Source: PLoS One. 2022 Apr 7;17(4):e0264509. doi: 10.1371/journal.pone.0264509 (PMC8989295; doi:10.1371/journal.pone.0264509)
Supplement: Attachment — Submitted filename: response to reviewers PlosOne R2.pdf [file pone.0264509.s008.pdf]

Reviewer #1: The authors have revised and resubmitted their manuscript describing ETC models to evaluate the effects of cultural transmission and environmental factors on cultural change. I liked the paper on its initial submission and find it to be considerably improved following the authors' revisions. I thank the authors for their work following the previous round of review, and I now consider the manuscript suitable for publication, although I will note two minor points:

line 32 – I appreciate the authors removal of the word institutions here, but there remains an issue with wording. As it stands, the authors now seem to be putting various features of the environment within "culture", for instance pathogen presence. However, this is not typical. For instance, environmental features that arose independently of human actions might nonetheless influence how we behave, yet to include these features themselves as "culture" is unusual because it is not clear how they are being inherited. To the extent that human action has created environmental features (e.g. global warming) it is common to talk of them being inherited, but this would normally be placed within niche construction or ecological inheritance, not necessarily cultural inheritance. More generally, I think the authors need to recognize that at one extreme their model does not concern culture at all – if their model found that transmission had no role, and all that mattered was environmental state, then the process at work is simply one of adaptive phenotypic plasticity and not culture. To resolve this the wording of the manuscript needs tweaking, particularly in this section, though the authors might spot other places to make useful changes too. The wording is great, for instance, in lines 100-101 where the point of the model is described as identifying the roles of transmission and ecology, and I would suggest that the latter is not part of culture per se (though it can of course influence culture).

The reviewer is right when writing "To the extent that human action has created environmental features (e.g. global warming) it is common to talk of them being inherited, but this would normally be placed within niche construction or ecological inheritance, not necessarily cultural inheritance." Our model was not made to compare ecological inheritance and informational inheritance, but only ecology and transmitted information. The last sentence of the paragraph is thus not relevant here, and we have decided to remove it and replace it by:

> *"Here we are interested in quantifying the relative influence of evoked and transmitted forces onto cultural evolution."*

line 73 – typo? "action facial unites"?

Thank you for noting this, the correct expression is "facial action units"
Ekman, P., & Rosenberg, E. L. (Eds.). (1997). *What the face reveals: Basic and applied studies of spontaneous expression using the Facial Action Coding System (FACS)*. Oxford University Press, USA.

Reviewer #2: Thank you for addressing all the points raised in my first review. I have two minor comments on the revised manuscript and one optional additional suggestion.

Line 208: Why doesn't the cultural trait converge to simply the default? In other words, why does rho still appear in the denominator?

This is simply a matter of the how the linear model is defined. Using the current definition, solving equation (6) without noise, ecological variable and connected region gives that the stable point is at $-\rho T_{t,r} + \gamma = 0$, i.e. $T_{t,r} = \rho/\gamma$.

We could have used another parametrization for equation (6) of the form $\frac{dT_{t,r}}{dt} = -\rho(T_{t,r} - \tilde{\gamma}) + \cdots$, in other words using the reparametrization $\tilde{\gamma} = \rho/\gamma$. Here this changed parameter $\tilde{\gamma}$ would be directly defined as the "default trait value". The two systems are equivalent, really.

Line 211: I hope it is a Wiener process and not a Wiener, an inhabitant of the city of Vienna. Or is it the *other* wiener and I completely misunderstood the point of transmitted culture? :D

Thanks for pointing the missing word. That alternative hypothesis about the source of stochasticity in cultural evolution will be the subject of a subsequent paper.

Initial comment on the presentation of the model

The authors now explain all model parameters, but they have not addressed my central issue and neither improved the presentation of the model nor justified the design decisions. For example, why is cultural lability a necessary ingredient of the equation? Why do the external factors matter?

I don't want to stay in the way of the paper getting published because I see the merit of the work. Still, I would encourage the authors to explain the generative story of their model first and then provide the equations, which will allow the readers to judge the model's relevance for their problems and increase its adoption in the community. I leave it to the authors whether or not they want to address this point.

We thank the reviewer for helping us find how the clarity of the model presentation could be improved. We have made another attempt to try to improve it. It is about the best we can do, I am afraid. The following has been added to the manuscript (additions are underlined).

> *We propose a simple stochastic linear model for the dynamics of cultural traits, where the degree of vertical transmission, horizontal transmission and evoked component (i.e. influence of the ecological variable) are controlled by separate parameters (to be estimated from the data). (…)*
>
> *Parameter ρ is the cultural lability (or cultural leak). It determines the time scale at which the memory of cultural traits is lost, in other words it controls the degree of vertical transmission.*
>
> *Because the raw value of cultural traits is usually arbitrary and one is usually mostly interested in relative values, one can often assume that γ =0, i.e. that the "default" trait value is null. If this is not the case, one must take caution in defining the generative process for artifacts to make sure that a bias term is not present that would make parameters not identifiable. For example, for binary artifacts, one could use $p(A_{t,r}) = S(\beta_0 + \beta_1 T_{t,r})$ if γ is assumed to be null or $p(A_{t,r}) = S(\beta_1 T_{t,r})$ otherwise; the two systems are*

mathematically equivalent, they only differ in whether the constant bias β_0 is absorbed into a fixed offset of cultural traits or not.

The term "external factor" was not adapted, we actually referred to the ecological variable: λ controls the importance of the importance of the evoked component. This has been changed in the manuscript, and we hope it is clearer now.
